# Evolutionary Psychology and Normal Science: in Search of a Unifying Research Program

**DOI:** 10.1007/s12124-022-09736-x

**Published:** 2022-12-07

**Authors:** Jonathan Egeland

**Affiliations:** University of Agder, Kristiansand, Norway

**Keywords:** Evolutionary psychology, normal science, research program, controversy

## Abstract

Why are there so many controversies in evolutionary psychology? Using a couple of concepts from philosophy of science, this paper argues that evolutionary psychology has not reached the stage of mature, normal science, since it does not currently have a unifying research program that guides individual scientists working in the discipline. The argument goes against claims made by certain proponents and opponents of evolutionary psychology, and it is supported by discussion of several examples. The paper notes that just because evolutionary psychology has not reached the stage of normal science, the discipline is nevertheless a source of many progressive theoretical developments and interesting empirical discoveries.

## Introduction

The purpose of evolutionary psychology is to understand human traits and behaviors as products of evolved psychological mechanisms that improved our ancestors’ chances of reproducing and/or surviving. Evolutionary psychology takes Darwin’s theory of natural selection as its point of departure, claiming that ancestral populations characterized by phenotypic variation, heredity and differential fitness evolved a great number of psychological adaptations that were conductive to individual reproduction and/or survival.^1^ In other words, using evolutionary theory, it provides putative explanations of psychological mechanisms; sometimes these explanations only focus on *ultimate causes*—which is to say that they are distal explanations of *why* our psychological mechanisms exist—and other times they also focus on *proximate causes*, which concern *how* psychological mechanisms work.^2^

However, the discipline remains controversial, with some considering it “an indispensable, not optional, ingredient for a mature psychological science” (Confer, Easton et al. 2010, 111), and others claiming that it is “a deeply flawed enterprise” (Dowens, 2021) and “wrong in almost every detail” (Buller 2005).^3^ This paper will use a couple concepts from philosophy of science in order to explain why there is so much disagreement about whether, or to what extent, evolutionary psychology is a scientifically legitimate enterprise. Indeed, it will argue that the main cause of controversy is the fact that evolutionary psychology has not reached the stage of mature, *normal science*, but that it rather is a battleground of competing *research programs* with different theoretical foundations.^4^ Moreover, the paper notes that although evolutionary psychology is not at the moment a mature, normal science, it has nevertheless led to numerous progressive theoretical developments and interesting empirical discoveries (cf. Lukaszewski, Lewis et al. 2020, 4); indeed, it provides the only viable naturalist framework for understanding human nature and behavior as the biological phenomena that they are.

The paper has the following structure. The second section introduces and explains the concepts of “normal science” and “research program”. The third section claims that many of the disagreements that exist with respect to evolutionary psychology and its scientific legitimacy are symptomatic of the fact that there currently is no single, unifying research program that most researchers adhere to. The fourth section supports this claim by discussing six fundamental issues about which researchers working on conceptual or empirical issues in evolutionary psychology generally disagree: modularity, adaptationism, human nature, ongoing evolution in modern humans, group selection, and novelty adaptations. The last section discusses the paper’s argument, and it offers some reflections on the future of evolutionary psychological science.

## What is Normal Science?

In his work on *The Structure of Scientific Revolutions* (2012), Kuhn argues that the process of scientific change occurs in a cyclical and stepwise fashion. It starts off in a state of relative disorganization, in which there is little or no consensus on any theory. Once a theory provides the basis for important discoveries, then normal science can begin as researchers stop debating fundamentals and start “puzzle-solving”—i.e., they try to solve new conceptual or empirical puzzles by using the theoretical foundation (about which there is a consensus opinion that it is correct, or at the very least that it is to be pursued) to make sense of them. However, if anomalies that should be solvable accrue, then the scientific community enters a crisis that either is resolved by continuing the work of normal science, or it results in a scientific revolution that eventually leads to the adoption of a new theoretical foundation that allows scientists to return to normal science.

Kuhn calls the theoretical foundation that characterizes normal science a *paradigm*. However, there are good reasons to prefer Lakatos’s notion of a *research program* instead. According to Lakatos, scientific theories are not evaluated in isolation, but rather as parts of a larger research program to which they belong. Indeed, a research program is constituted by a sequence of theories that all share the same *hard core* of theses that are, due to the research program’s *negative heuristic*, made “‘irrefutable’ by [a] methodological decision of its proponents” (Lakatos 1978, 48). The hard core is made irrefutable in the sense that it does not in and of itself provide any deducible empirical predictions and, moreover, rejecting it is invariably considered a rejection of the research program that it belongs to. Furthermore, the individual theories comprising a research program all rely on certain *auxiliary hypotheses* that allow for the derivation of predictions and that differentiate them from each other. Whenever a prediction fails to be corroborated by the empirical data, it is (some of) the auxiliary hypotheses of the theory from which the prediction was derived, rather than the hard core, that are falsified (which is why Lakatos names them the *protective belt*). Moreover, when this happens, the research program also has a *positive heuristic* consisting of a “partially articulated set of suggestions” about how the auxiliary hypotheses are to be modified, in order to ensure that the latest and most sophisticated theoretical development within the research program has not been refuted by the empirical evidence (Lakatos 1978, 50).

There are primarily two reasons why this paper focuses on Lakatos’s notion of a research program, rather than Kuhn’s notion of a paradigm. The first is that Lakatos’s notion is part of a much more sophisticated conceptual framework that provides a philosophical account of changes within individual research programs, as well as a way of evaluating competing research programs.^5^ The second is that Lakatos’s conceptual framework is increasingly being used in the psychological research literature in order to understand and evaluate different hypotheses or theories therein. For example, it has been used in the debate about individual and group differences in general intelligence (Urbach 1974, Rushton and Jensen 2005),^6^ in the context of secular trend analysis (Egeland, 2022), and in the discussion about falsifiability in evolutionary psychology (Ketelaar and Ellis 2000).^7^ Continuing, the next section will use the concepts of normal science and research program to diagnose why exactly there is so much controversy and disagreement when it comes to the merits and demerits of evolutionary psychology.

## Evolutionary Psychology (Currently) has no Unifying Research Program

The core thesis of this paper is that evolutionary psychology has not (yet) reached the stage of mature, normal science, since there is no unifying research program that guides individual scientists working in the discipline. Rather, what we find is that evolutionary psychology is characterized by numerous competing research programs, and that there is not (at the moment) any such program that appears likely to achieve the status of consensus opinion.^8^ However, this is a thesis that both proponents and opponents of evolutionary psychology have explicitly argued against.

In their defense of evolutionary psychology as a progressive Lakatosian research program, Ketelaar and Ellis (2000) argue that the discipline *has* a unifying research program that guides scientists working to understand the ultimate causes of human behavior. They claim that the hard core consists of a metatheory, which is “a set of consensually held basic assumptions that shape how scientists generate, develop, and test middle-level theories and hypotheses”, and that “in the case of evolutionary psychology, the metatheoretical level consists of the general principles of genetical evolution drawn from modern evolutionary theory” (Ketelaar and Ellis 2000, 4). So, in other words, the hard core of the research program that is evolutionary psychology consists of the core tenets of the theory of evolution by natural selection.

But what about the protective belt—i.e., the auxiliary hypotheses of the research program? The protective belt, Ketelaar and Ellis (2000, 6) tell us, is broken down into three levels of analysis: middle-level theories, hypotheses, and predictions. Middle-level theories (such as Trivers’s (1972) parental investment and sexual selection theory) are consistent with the hard core, and they provide inferential links to specific hypotheses from which testable predictions can be derived. (cf. Buss 2019, ch. 2, who appears to assume a similar view in his comprehensive introduction to evolutionary psychology.)

The problem with Ketelaar and Ellis’s understanding of evolutionary psychology is that their conception of its hard core is too broad. By arguing that the hard core consists of the general principles of evolution by natural selection, it follows that evolutionary psychology is just a very small part of a vast research program that includes most branches of both psychology and biology. Indeed, any theory in any field that includes the theory of evolution by natural selection in its theoretical foundation will by definition belong to the same research program as evolutionary psychology, since any two theories that share the same hard core invariably belong to the same research program. However, it is not just implausible that evolutionary psychology belongs to the same research program as, say, contemporary molecular entomology, but the consequences of this position become downright absurd as one would have to include theoretical approaches that are in direct conflict with standard evolutionary psychology, such as Gould & Lewontin’s (1979) approach focusing on the relative importance of *spandrels* and *exaptations*^9^ rather than adaptations, in the same research program.

Moreover, another problem with Ketelaar and Ellis’s position is that it makes it very hard to understand why exactly there is so much controversy surrounding evolutionary psychology. If any theory in any scientific field that includes the theory of evolution by natural selection in its theoretical foundation necessarily belongs to the same research program as evolutionary psychology, then why does evolutionary psychology stand out as a discipline about which there is a rather remarkable amount of both internal (from within) and external (from without) disagreement? There does not appear to be a forthcoming answer, as long as one assumes that Ketelaar and Ellis’s position is correct. After all, a basic Lakatosian idea is that researchers working within the same research program are in agreement about the fundamentals of their discipline. Furthermore, when there is a dominant research program that guides the work of the majority of researchers in a certain discipline, then they have reached the Kuhnian stage of normal science. However, when it comes to how evolutionary psychology actually is practiced, and to the various theoretical commitments that its practitioners actually take on, it is quite clear that there is no unifying research program in evolutionary psychology, and that the discipline has not reached the stage of mature, normal science. This is a point that will be illustrated using various examples in the next section.

Proponents of evolutionary psychology are, however, not alone in thinking that the discipline has a unifying research program. Indeed, this is a claim voiced by its opponents also. Buller (2005), and others following him (e.g., Dowens 2021), draw a distinction between “evolutionary psychology” (henceforth referred to as “ep”) that encompasses evolutionary approaches to human behavior and mind in general, and (the capitalized phrase) “Evolutionary Psychology” (henceforth referred to as “EP”) that is committed to certain very specific theoretical theses associated with the *Santa Barbara School* (or so-called “High Church”, Heyes 2012). The theoretical foundation of EP has been articulated in different ways, and the following characterization by the Santa Barbara psychologists John Tooby and Leda Cosmides is one way of doing so:^10^

1. The brain’s evolved function is to extract information from the environment and use that information to generate behavior and regulate physiology[… The brain] is a computer—that is, a physical system that was designed to process information. Its programs were designed not by an engineer, but by natural selection, a causal process that retains and discards design features based on how well they solved adaptive problems in past environments[…]^11^
2. Individual behavior is generated by this evolved computer, in response to information that it extracts from the internal and external environment[…] To understand an individual’s behavior, therefore, you need to know both the information that the person registered *and* the structure of the programs that generated his or her behavior.^12^
3. The programs that comprise the human brain were sculpted over evolutionary time by the ancestral environments and selection-pressures experienced by the hunter-gatherers from whom we are descended[…].
4. Although the behavior our evolved programs generate would, on average, have been adaptive (reproduction promoting) in ancestral environments, there is no guarantee that it will be so now[…] Each evolved program exists because it produced behavior that promoted the survival and reproduction of our ancestors better than alternative programs that arose during human evolutionary history. Evolutionary psychologists emphasize hunter-gatherer life because the evolutionary process is slow—it takes thousands of generations to build a program of any complexity. The industrial revolution—even the agricultural revolution—is too brief a period to have selected for complex new cognitive programs.^13^^,^^14^
5. Natural selection will ensure that the brain is composed of many different special purpose programs, many (or all) of which will be specialized for solving their own corresponding adaptive problems. That is, the evolutionary process will not produce a predominant general-purpose, equipotential, domain-general architecture[…]^15^
6. Descriptions of the computational architecture of our evolved mechanisms allow a systematic understanding of cultural and social phenomena[…]^16^ (Tooby & Cosmides, 2005, 16–18)

On this view, the aforementioned theses constitute the hard core of the EP research program.^17^ (The auxiliary hypotheses are found in more specific theories/hypotheses that develop and build upon this hard core, and that (often) offer testable predictions.) However, there are a couple of reasons as to why the claim that there is a unifying EP research program is problematic.

First, (just like with Ketelaar and Ellis’s position) its conception of the hard core is too broad (*cf*. Zagaria, Ando’ et al. 2020), since some of its central theses are shared by proponents of ep too. For example, Buller (2005, 200) takes issue with the adaptationist thesis (nr. 3) above, claiming that the mind’s psychological mechanisms “weren’t shaped by selection over our species’ evolutionary history”. However, this claim is certainly false—for a discussion with examples, see Machery and Barrett (2006, 233–234)—since virtually every scientist studying human behavior from an evolutionary perspective will concede that many of the psychological mechanisms of the human mind are evolutionary adaptations. Moreover, as the hard core of EP is broad enough to include theses that also are endorsed by proponents of ep, this may raise legitimate concerns as to whether the dichotomous ep-EP distinction really is adequate (*cf*. Machery and Barrett 2006, 233–234).

Second, the hard core of the EP research program may paradoxically in some sense also be too narrow. The purpose of the ep-EP distinction is to show that there indeed is a unifying research program in evolutionary psychology (namely, the EP research program) that is distinct from other evolutionary approaches (referred to as ep) to the human mind and human behavior. However, since many evolutionary psychologists do not subscribe to the hard core articulated by proponents of the Santa Barbara School (or to similar permutations thereof), it follows that the hard core does not provide the basis for a unifying research program. (As previously mentioned, this is something that will be discussed in greater detail in the next section.) Indeed, claiming that all of evolutionary psychology operates within the EP research program, and that the faults of EP necessarily undermine evolutionary psychological science in general, is no more true than claiming that all of social psychology operates within the theoretical framework of the research on priming effects, or that potential faults of the latter threaten to undermine social psychology in general.

## Fundamental Points of Disagreement Among Evolutionary Psychologists

Having presented some problems with the view that there is a unifying research program in evolutionary psychology, this section will offer support for the opposing perspective—according to which the discipline has not reached the Kuhnian stage of normal science—by showing that there are several fundamental points of disagreement among its practitioners. The examples discussed in the current paper are summarized and presented in Table 1. More specifically, the idea is that the (non-ideological) controversy and disputes that evolutionary psychology clearly engenders can be explained as a consequence of the fact that the practitioners of the discipline disagree about fundamental theoretical issues, such as the structure of human psychological mechanisms, the theoretical and conceptual assumptions underlying adaptationist explanations, and also the very nature of how evolutionary processes function as they sculpt our heritable phenotypes.

**Table 1:** Examples of fundamental theoretical disagreements in evolutionary psychology

Issues	Central questions	Examples of commentators with different perspectives
Massive modularity	Are there domain-general psychological mechanisms in the human mind?	Tooby and Cosmides (2000), Bolhuis, Brown et al. (2011).
Adaptationism	What is the role of epigenetics in adaptationist reasoning?	Gregory (2009), Tooby & Cosmides (2015).
Human nature	Is there a single, species-typical human nature, or is there a plurality of human natures?	Winegard, Winegard et al. (2017), Lukaszewski (2021).
Ongoing evolution in modern humans	To what extent has there been adaptive evolution in modern human populations?	Cosmides & Tooby (1987), Chekalin, Rubanovich et al. (2019).
Group selection	Does natural selection operate on human groups?	Sober & Wilson (1998), Dawkins (2006), Pinker (2018).
Novelty adaptations	Are there psychological adaptations to environmental novelty?	Barrett and Kurzban (2012), MacDonald and Woodley of Menie (2016).

### Massive Modularity

A core thesis of the Santa Barbara school is that the human mind consists of a large number of mental modules, which are domain-specific cognitive subsystems that have evolved due to their adaptive function in the EEA:

From an evolutionary perspective, the human cognitive architecture is far more likely to resemble a confederation of hundreds or thousands of functionally dedicated computers […] than it is to resemble a single general purpose computer equipped with a small number of domain-general procedures (Tooby and Cosmides 2000, 1171).^18^

In other words, the massive modularity thesis says that our cognitive architecture can be likened to that of a Swiss army knife, insofar as both have a number independent designs that each serve to solve specific kinds of problem (Pinker, 1995).

Several arguments have been forwarded in defense of this thesis, such as that “[t]here is no such thing as a ‘general problem solver’ because there is no such thing as a general problem” (Symons, 1992, 142), and that a domain-general mechanism that serves to solve adaptive problems cannot have evolved since it would (in the absence of any domain-specific procedure) have to evaluate all conceivable behavioral solutions, which would be too time consuming for practical purposes (due to combinatorial explosion) and, hence, ultimately leave the individual paralyzed (Cosmides and Tooby 1994, 94).

However, despite these (and other) arguments,^19^ proponents of massive modularity no longer seem to have the upper hand against their dialectical opponents. There are a number of reasons for this, some of which function as responses to the arguments briefly mentioned above, and others that also provide support for the idea that the human mind does have domain-general mechanisms or systems. For example, although our ancestors faced different adaptive problems, it does not follow that the solutions had to be implemented by independent domain-specific cognitive subsystems; rather, it is possible that a small number of domain-general mechanisms that are provided with domain-specific input (such as input from the visual system) can offer adaptive solutions (e.g., Samuels 1998, 587). Moreover, it is not necessarily the case that domain-general mechanisms will hamper someone’s fitness due to the computational complexity it faces, since a proper analysis of what a domain-general mechanism really is will open up for the possibility that domain-general mechanisms can solve different domain-specific problems in a timely and efficient manner by leveraging and coordinating the actions of relevant domain-specific subsystems. Indeed, this is plausibly how the immune system functions (Wilson, 2003, 30–31, Buller 2005, ch. 4).

The biggest problem for the massive modularity thesis is, however, that much of the empirical evidence indicates that the human mind has several domain-general problem-solving mechanisms. For example, one of psychology’s most replicated findings is that seemingly independent indicators of (say) cognitive ability or personality are both highly heritable (when properly measured) and correlated with each other, such that a general factor of intelligence (*g*) and a general factor of personality (GFP) typically explain more of the variance among individuals in these traits than any other relevant factor (Jensen, 1998, Chiappe and MacDonald 2005, Musek 2007, de la Fuente et al., 2019). Moreover, associative learning and memory apparently function in a domain-general manner in both human and non-human animals, as these mechanisms allow the organism to learn and remember causal relationships between a number of different events in widely varying contexts (Bolhuis and Macphail 2001, Lefebvre and Bolhuis 2003, Reader, Hager et al. 2011).^20^ That said, the point of this section is not to argue either for or against the massive modularity thesis, but rather to show that it constitutes one fundamental point of contention in evolutionary psychology.^21^

### Adaptationism

Evolutionary psychologists are generally committed to adaptationism, which claims that at least some psychological traits are adaptations for solving evolutionary problems faced by our ancestors. Indeed, evolutionary psychology has inherited from evolutionary biology and sociobiology the adaptationist principle that “many psychological characteristics are adaptations—just as many physical characteristics are—and that the principles of evolutionary biology that are used to explain our bodies are equally applicable to our minds” (Durrant and Ellis 2003, 5). Now, there are many disagreements when it comes to adaptationist thinking, but the one that I will briefly mention here has to do with the idea that biologically inherited psychological adaptations have their etiological roots in genetic evolution alone.

It is not uncommon for adaptationists to assume a gene-centered view of evolution. For example, as Cronin (2005, 19–20) notes: “The purpose of adaptations is to further the replication of genes […] Genes have been designed by natural selection to exploit properties of the world that promote their self-replication; genes are ultimately machines for turning out more genes”. However, recent findings may perhaps challenge certain adaptationist assumptions that take for granted the gene-centered view of evolution. One such example is the phenomenon of transgenerational epigenetic inheritance, whereby heritable phenotypic changes occur, but without any change in DNA sequence (Heard and Martienssen 2014). Empirical support for the phenomenon has led some evolutionary psychologist to argue that cross-generational effects of epigenetic inheritance ought to be considered evolved adaptations, and that personality variation may be produced by calibrational epigenetic systems that use certain developmental cues as inputs (Tooby & Cosmides, 2015, 75–79).

Similarly, the discovery that an individual’s adaptive psychological mechanisms may be influenced not just by their own genotype, but by the genotypes of other conspecifics, indicates that our conceptual framework for dealing with behavioral phenotypes and their genotypic bases may need revision (see Domingue et al., 2018, Kong, Thorleifsson et al. 2018, for more on indirect genetic effects (IGEs)). Although the occurrence of IGEs does not threaten the gene-centered view underlying much of current adaptationist thinking, it nevertheless demonstrates that we need a refined conceptual framework for adequately understanding the complex relationships between genotypes and psychological adaptations. Just as Dawkins’ (2016) idea of the extended phenotype has been very useful for theorizing about evolution (consider the example of niche construction, whereby an organism’s genetically influenced behavior leads to an alteration in the environment), it may be time for evolutionary psychology to start focusing on organisms’ extended genotypes and their role in the development or activation of adaptive psychological mechanisms.

### Human Nature

Some evolutionary psychologists believe that the psychological mechanisms of the human mind that have evolved as a response to adaptive problem-solving in our ancestral past constitute a universal, species-typical human nature. These psychological mechanisms are taken to be “universal among *Homo Sapiens*” (Symons, 1992, 139), which is to say that they are psychological universals constituting a “human nature [that] is everywhere the same” (Tooby and Cosmides 1992, 38). On this view, individual differences in psychological adaptations are typically considered random variation resulting from “genetic noise” around a “species-typical” mean level of the adaptations in question (Tooby and Cosmides 1990), or environmentally mediated patterns of differential mechanism activation with variable cost-benefit tradeoffs depending on the organism’s individual or social ecological context (Lukaszewski 2021).

However, some evolutionists, like Penke, Denissen et al. (2007), Winegard, Winegard et al. (2017), argue that this universalist view conflicts with the fact that many psychological traits are moderately or highly heritable (for relevant evidence, see Polderman, Benyamin et al. 2015, Plomin, DeFries et al. 2016), meaning that much of individual trait variation can be explained by genotypic variation (Egeland 2023). In response to this comparatively pluralist view, Tooby and Cosmides (1990), Lukaszewski (2021) argue that it involves a conflation of “deep” and “manifest” structures of psychological adaptations, since it only is the latter that (whether due to genetic or environmental proximate causes) can display systematic intraspecific variation. Now although this response may be successful in undermining many of the arguments forwarded by those who endorse the pluralist position regarding human nature (insofar as they conflate deep and manifest structures), it nevertheless fails to undermine said position due to the question-begging nature of the response. Why does it beg the question? The reason is that the concepts of deep and manifest structure are defined in terms of intraspecific invariance and intraspecific variance respectively (cf. Lukaszewski 2021), meaning that it assumes the correctness of the conclusion that there is a universal, species-typical human nature with respect to the deep structure of our psychological adaptations. However, the issue of human nature and the deep structure of our psychological adaptations—i.e., universalism vs. pluralism—cannot be settled by *a priori* definition, but has to be evaluated on the basis of empirical evidence and what we know to be true of evolutionary theory.

Despite their disagreements regarding human nature, evolutionary psychologists are increasingly working to develop new models of personality and other traits that display large, heritable individual differences (e.g., MacDonald 1995, Wilson, Near et al. 1996, Figueredo et al., 2005, Lukaszewski, Lewis et al. 2020).^22^ For an overview of some promising theoretical approaches to a systematic evolutionary psychological understanding of individual psychological differences, see Buss (2009). Moreover, regardless of how these debates turn out, it is nevertheless noteworthy that there still is so much disagreement about human nature and the importance of individual differences among evolutionary psychologists.

### Ongoing Evolution in Modern Humans

Some proponents of the EEA concept (cf. footnote 14 above), including the Santa Barbara school, consider evolution to be a rather slow process, occurring on a relatively large timescale, and they argue that our psychological adaptations evolved when our ancestors were in the EEA, sometime during the Pleistocene. One consideration sometimes invoked to support this position is that our species spent most of its time in the Pleistocene, before the introduction of agriculture and animal domestication: “Our species spent over 99% of its evolutionary history as hunter-gatherers in Pleistocene environments. Human psychological mechanisms should be adapted to those environments, not necessarily to the twentieth-century industrialized world” (Cosmides & Tooby, 1987, 280; cf. thesis 4 in Sect. 3 above).

However, this position has become increasingly controversial during recent years, as a large number of genes have been affected by evolutionary processes since we “left” the EEA (Williamson, Hubisz et al. 2007), with some evidence indicating that the evolution of human psychological phenotypes actually may have sped up during the Holocene when modern humans adopted agricultural practices (Hawks et al., 2007, Laland, Odling-Smee et al. 2010). Indeed, some novel traits essentially came “online” sometime during last 10 000 years (such as the continued production of lactase, due to the domestication of cattle: Cochran & Harpending 2009, 77), and there is even evidence that natural selection has occurred with respect to a large number of psychological traits during the last century (Clark et al., 2014, Chekalin, Rubanovich et al. 2019, Hugh-Jones and Abdellaoui 2021).^23^

Much of the recent evolution that has occurred during the Holocene is plausibly a result of genetic and cultural interaction, as explained by the theory of culture-gene coevolution (Cavalli-Sforza & Feldman, 1981; Boyd & Richerson, 1988; Lumsden & Wilson, 2005). The theory posits that cultural practices change as a result of changes in a population’s genes. But once a new cultural practice is established, it creates selection pressures that may open up new evolutionary spaces and lead to novel adaptations.^24^ However, the resultant changes in gene frequencies may spur the development of even newer cultural practices, and so on as genes and culture continually interact and create a feedback loop that speeds up the evolutionary process. Cochran & Harpending (2009), Bolhuis, Brown et al. (2011) provide several examples of such culture-gene coevolution, and recent data indicate that cultural complexification characteristic of the Holocene has sped up adaptive evolution in certain populations (Richerson and Boyd 2005, Hawks et al., 2007, Laland, Odling-Smee et al. 2010).

### Group Selection

There is a lot of disagreement among evolutionary psychologists as to whether evolution only operates at the level of the individual, or whether it also operates at the group level. In order to get a grip on where exactly the disagreement lies, it is necessary to differentiate the *unit of selection* from *levels of selection*. In the case of genetical evolution, the unit of selection is individual genes that produce copies of themselves. Moreover, genes interact with their environments in order to influence their own survival, as well as that of their copies, and their influence is manifested (via the process of natural selection) at the level of individual organisms and, some argue, at the level of groups (and perhaps other levels also). Using Dawkins’s (1978, 2006) distinction between *replicators* and *vehicles*, we can say that the unit of selection is genes that function as replicators, whereas organisms (and, perhaps, the groups they comprise) are vehicles in which the replicators travel about, and on which they exert their fitness-enhancing influence in order to ensure their own and their copies’ survival.^25^

Some evolutionary psychologists and proponents of adaptationist reasoning in general have adamantly argued that the only level at which selection acts is the individual organism. For example, Dawkins (2006) and Pinker (2018) strongly argue against group selection, which the latter “contrasts with mainstream evolutionary psychology, in which the unit of selection is the gene” (Pinker, 2018, 448). However, a problem with this particular piece of reasoning is that it appears to erect a *straw man* that easily can be knocked down by referencing the generally agreed upon proposition that it is the gene (and not the group) that is the unit of selection. However, as contemporary proponents of multilevel selection theory argue that groups (and other levels of biological organization) under certain conditions constitute a *level* at which selection can act, the aforementioned critique may very well rest on a conflation of units and levels of selection in its presentation of the group selectionist position (Okasha, 2006, 13–18).

One of the strengths of group selection is that it is able to explain why certain types of behavior in both human and non-human animals have not been eliminated by natural selection, even though they incur a fitness cost to the individual animal. A clear example of this is altruism, which can be defined as any behavior that somehow benefits some other organism, while at the same time reducing the likelihood that the animal that acts altruistically will reproduce. Darwin and other group selectionists following him have argued that a group containing altruists that are prepared to behave in a manner that is detrimental to their own fitness but for the good of the group, may have an evolutionary advantage over groups without such members—which means that group selection can account for the Darwinian puzzle that is the existence of altruists: “a tribe including many members who […] were always ready to give aid to each other and sacrifice themselves for the common good, would be victorious over most other tribes; and this would be natural selection” (Darwin, 1871, 166). This may perhaps be why multilevel selection theory is seeing an increasing number of adherents in the scientific community (Yaworsky, Horowitz et al. 2015). However, a potential problem with the group selectionist explanation is the existence of free-riders who exploit the altruists by consistently behaving in a selfish manner, and who therefore eventually should out-compete them in an evolutionary sense of the of term.^26^ Briefly put, the issue of group selection in general, and altruistic behavior in particular, continues to puzzle evolutionary psychologists, who still pursue different models and theoretical approaches to the understanding of seemingly groupish psychological traits.

### Novelty Adaptations

The last source of disagreement that I will mention is what may be called *novelty adaptations*, which are evolutionary adaptations to environmental novelty. Some evolutionary psychologists have argued that novelty adaptations cannot exist, since a necessary condition for an adaptation is the recurrence of some relevant environmental signal:

It is only those conditions that recur, statistically accumulating across many generations, that lead to the construction of complex adaptations […] For this reason, a major part of adaptationist analysis involves sifting for these environmental or organismic regularities or invariances (Tooby and Cosmides 1992, 69).

[A]t a certain level, the terms “design” and “novelty” are incompatible with each other, because adaptation is impossible without *some* environmental signal, even if statistically fuzzy, to adapt to. If “novel” means “bears no resemblance to anything in the past,” then design to deal with novelty is a priori impossible (Barrett and Kurzban 2012, 686).

However, some evolutionary psychologists have criticized this position (e.g., Potts 1998), arguing instead that certain psychological traits, like general intelligence, indeed are novelty adaptations. For example, Kanazawa (2012) hypothesizes that human general intelligence is a domain-specific adaptation that was selected for when humans migrated out of the evolutionarily familiar African Savanna, and he predicts that behaviors that are in some sense “evolutionarily novel” will correlate with IQ. However, Kanazawa’s hypothesis has a number of problems, such as that human intelligence does not appear to be domain-specific, it arguably relies on an outdated conception of the EEA, and the hypothesis allows for the derivation of contradictory predictions (Penke, Borsboom et al. 2011, Dutton 2013).

In a recent review of the literature on the evolution of intelligence, MacDonald and Woodley of Menie (2016) synthesize finding from a large number of disciplines—including psychometrics, evolutionary biology, neuroscience, and animal intelligence research—arguing that the most coherent perspective on the evolution of general intelligence sees it as a domain-general adaptation that interacts with motivational mechanisms that provide the organism with information about whether certain evolutionary problems whose solutions are underspecified (i.e., novel) have been solved, by inducing in the organism positive or negative subjective feelings:

The affective basis of domain generality is evolutionarily ancient, resulting primitively in simple associative learning mechanisms (classical and operant conditioning), then elaborated greatly with social learning, and finally general intelligence as a suite of mechanisms, particularly the executive process of working memory […] underlying the ability to manipulate information from a variety of sources in order to achieve goals that may or may not be linked with affective motivational systems derived from the evolutionary past. (MacDonald and Woodley of Menie 2016, 2547).

Now although this is a coherent hypothesis that is consilient with the latest research in various disciplines (especially if it is combined with the idea that a distal cause of human general intelligence, as well as a number of other traits that are unique to our species, is runaway social selection: Alexander 1989, Flinn, Geary et al. 2005, Crespi et al., 2022), the idea that there really is such a thing as adaptations to environmental novelty remains controversial. Indeed, this is just one example of how evolutionary psychology is mired in controversy and disagreements, and as the examples discussed above illustrate, a plausible explanation of this fact is that evolutionary psychology has not reached the Kuhnian stage of mature, normal science, in the sense that there is no unifying research program that guides its individual researchers. Rather, what we find is that evolutionary psychologists *systematically* disagree about fundamental theoretical issues, at least some of which need to be resolved before *progress that is recognized as progress* by both those who work within the discipline and by those who are outsiders looking in can be made.

## Discussion

This paper has argued that the primary cause of (non-ideological) controversy in evolutionary psychology is that the discipline does not have a unifying research program, which means that it has not reached the stage of normal science. This is evidenced by the fact that evolutionary psychologists disagree about a large number of fundamental theoretical issues, such as the structure of human psychological mechanisms, the importance of the gene-centered perspective in adaptationist explanations, and the very nature of how evolutionary processes function as they sculpt our heritable phenotypes.

Another potential source of controversy, however, is the methods or inferential strategies used by evolutionary psychologists in order to arrive at plausible adaptationist explanations. In a nutshell, one starts by identifying adaptive problems that our ancestors likely faced, and from this one infers hypotheses about which psychological mechanisms must have evolved to solve these problems. The hypotheses are then evaluated by testing their predictions (or by seeing how much of the relevant data they can explain), and by comparing them to other, non-adaptationist hypotheses.^27^ However, this type of reasoning has been subject to fierce criticism, the most prominent of which has been articulated by Gould & Lewontin (1979, 43; cf. Pigliucci, 2010), who argue that evolutionary psychological hypotheses and theories are not really tested in a way that makes them vulnerable to falsification, but that they rather constitute “just-so-stories” that never can be established as facts.

There are two reasons why methodological objections of this kind are not particularly problematic for evolutionary psychology, and why this paper has focused on theoretical issues instead. The first reason is that it is neither true that all hypotheses in evolutionary psychology are not vulnerable to falsification, nor that they are all just-so-stories without any basis in fact. Just to give one example, the replicated finding that there are sex differences in romantic jealousy was discovered only *subsequent* to the development of hypotheses about evolved psychological mechanisms which predicted that sex-differentiated patterns should be observable (Symons 1979, Buss, Larsen et al. 1992). The second reason is that since scientific theories and hypotheses never can proven to be true with complete certainty, one must always ask how fruitful a certain scientific approach is compared to other competing approaches. And when it comes to alternative approaches to providing explanations of the ultimate causes of human behavior, they generally fare much less well. For example, Gould’s spandrel concept simply does not generate predictions in the way that adaptationist thinking (sometimes) does. Rather, spandrel-based explanations are usually only offered in the absence of some convincing adaptationist explanation—and in this sense it seems, ironically enough, much more fitting to consider those explanations just-so-stories.^28^

That said, there are certainly both good and bad theories in evolutionary psychology,^29^ and the discipline should not be rejected *tout court* just because some theory or research program therein is problematic. Moreover, my argument that evolutionary psychology has not reached the stage of normal science should not be interpreted as implying that it has the properties associated with Kuhn’s previous stage (the *pre-paradigm* stage), which would imply that there is almost no agreement at all in the discipline, and that there virtually is no progress being made. Although there is not at the moment any unifying research program in evolutionary psychology, one nevertheless finds fruitful and progressive theoretical developments being made, and it is certainly possible that the discipline will enter the stage of normal science in the near future.

## Footnotes

1 In *The Descent of Man*, Darwin (1871) himself argues that human “mental faculties” are adaptations produced by natural and sexual selection.

2 It should be noted that the different levels of analysis with respect to which different explanations of biological and psychological phenomena operate in some sense impose a certain normative structure on how we *ought* to conceptualize our understanding of human behavior. Theoretical developments and empirical findings at one level may be suggestive of how hypotheses at other levels should be formulated or tested, and failure to recognize that certain kinds of question only can be answered at a certain level of analysis may lead to false debates or verbal disputes. Moreover, the possibility of integrative, between-level approaches also has to be considered, especially in light of the reductionism-holism debate. For helpful discussions of some of these issues, see Tinbergen (1963), MacDougall-Shackleton (2011).

3 See also Fodor (2001), Higgs (2001), Richardson (2007), Bolhuis (2008), Bolhuis and Wynne (2009), Bolhuis, Brown et al. (2011), Woodley of Menie and Sarraf (2018).

4 I will primarily focus on controversies and disagreements that aren’t motivated by ideology. For more on political or ideological criticisms that assume a blank slate view of the human mind, see Pinker (2003), Jonason and Schmitt (2016), Buss and von Hippel (2018).

5 That said, Lakatos’s conceptual framework does (just like any other conceptual framework in philosophy of science) have certain problems. See, e.g., Musgrave (1976), Laudan (1977, 77–78), Egeland (2022).

6 It was actually Lakatos himself who persuaded Urbach to write on environmentalism from the perspective of Lakatosian philosophy of science. For more on this, see Lakatos & Feyerabend (1999, 348–349).

7 See also Meehl (1978, 1990), Newell (1990), who argue that Lakatos’s philosophy of science provides a better framework for understanding and evaluating psychological science, compared to more standard falsificationist philosophies, such as that of Popper (1959).

8 Again, I reiterate that this should not be read as a criticism of any particular theory in evolutionary psychology. There are indeed many such theories that are plausible, important and supported by the evidence. The claim that evolutionary psychology is not a normal science must be interpreted from the philosophical perspective of Kuhn (cf. Section 2), in which case it will be understood that the purpose of this claim rather is to note that there is a sense in which evolutionary psychology is a fragmented discipline, since it is not uncommon for different practitioners to take on incompatible theoretical commitments (cf. Section 4). In other words: the paper completely agrees with the statement of Dobzhansky (1973) that “nothing in biology [including human behavior] makes sense except in the light of evolution”—it’s just that we need a unifying research program to guide our understanding of human psychology.

9 Exaptation occurs when the evolutionary function of a phenotypic trait changes over time. A spandrel is a phenotypic trait that is a byproduct of some evolutionary process, rather than (say) a fitness-enhancing adaptation.

10 Some, such as Buller (2005), Bolhuis, Brown et al. (2011), Grossi, Kelly et al. (2014), Walter (IEP-article), characterize the theoretical foundation of EP in similar, but slightly different ways.

11 Tooby and Cosmides’s first thesis assumes a computational model of the mind that is inspired by the works of Putnam (1963), Fodor (1975, 1981).

12 Theses 1 and 2 serve to differentiate EP from sociobiological perspectives, such as that of Wilson (1975), that the Santa Barbara school believes have neglected the importance of psychological mechanisms: “ In the rush to apply evolutionary insights to a science of human behavior, many researchers have made a conceptual ‘wrong turn’, leaving a gap in the evolutionary approach that has limited its effectiveness. This wrong turn has consisted of attempting to apply evolutionary theory directly to the level of manifest behavior, rather than using it as a heuristic guide for the discovery of innate psychological mechanisms” (Cosmides & Tooby, 1987, 278–279).

13 *Cf.* Cosmides & Tooby’s (1997) famous statement that “Our modern skulls house a Stone Age mind”.

14 Theses 3 and 4 are closely related to the concepts of *the environment of evolutionary adaptedness* (EEA) (Bowlby, 1969) and *gradualism*. The former concept refers to the idea that human psychological mechanisms are evolved adaptations produced by selection pressures that existed in ancestral environments, such as the African Savanna during the Pleistocene (Tooby and Cosmides 1990, Barkow, Cosmides et al. 1992). However, (and this is related to the latter concept) since these psychological mechanisms are adaptations to the EEA, it follows that contemporary humans may experience an *adaptive lag* or *evolutionary mismatch*, in the sense our psychological mechanisms may no longer lead to the same adaptive outcomes (cf. Laland and Brown 2006, and Symons’s, 1990, 430, distinction between a trait that is an adaptation and a trait that is adaptive).

15 This is also known as the *massive modularity thesis*, which claims that our psychological architecture consists of a large number of domain-specific, modular mechanisms or programs.

16 This is a reductionist thesis—one that arguably isn’t essential to EP, as reflected in the re-edited version of Tooby & Cosmides’ (2015) paper—claiming that social phenomena in general can be adequately explained from an EP perspective.

17 Buller (2005, 10 ff.) uses the Kuhnian notion of a paradigm, rather than Lakatos’s research program. However, for reasons given in the second section, I have decided to consistently use the latter term throughout this article.

18 *Cf.* Barrett and Kurzban (2006), Carruthers (2006).

19 For other arguments, see the reviews by Dowens (2021), Walter (IEP-article).

20 For more on these, as well as other objections to the massive modularity thesis, see Bolhuis, Brown et al. (2011), Woodley of Menie and Sarraf (2018).

21 For an interesting contribution, arguing that the whole debate about modularity is spurred by the confusion of different levels of analysis, see Pietraszewski and Wertz (2022).

22 It is not just heritable individual differences that call for an evolutionary explanation, but between-culture variation in behavioral traits also does so. An interesting development in this area is the *niche diversity hypothesis* by Smaldino, Lukaszewski et al. (2019), according to which more diverse social and ecological niches produce diverging behavioral profiles designed to be especially adaptive in a subset of such niches. This is an environmentalist hypothesis of cross-cultural differences in personality, claiming that assortment and plasticity (not heredity) are the primary mechanisms accounting for behavioral variation; and its prediction that lower niche diversity is inversely correlated with covariation among personality factors at the nation-level has been corroborated and replicated (Durkee, Lukaszewski et al. 2022).

23 It should also be noted that Cosmides and Tooby’s inference in the quoted paragraph above is not entirely sound. The reason is that just because humans as a species spent most of its time in the Pleistocene, it does not follow that significant evolutionary changes cannot have happened during, say, the last 10 000 years (cf. Walter IEP-article).

24 Indeed, the emergence of new cultural practices may, as work on the Baldwin effect suggests, favor the selection of a general learning ability, rather than more fixed abilities or behavioral dispositions (Richards, 1989). Moreover, the evolution of a general learning ability is plausibly conducive to the development of novel and useful cultural practices, since the individual now has a greater capacity for learning new skills and behaviors.

25 See, e.g., Dawkins (2016, 126), who presents the debate between individual selection and group selection as concerning which biological levels of organization can function as vehicles of selection.

26 A possible solution to this problem of “subversion from within”, as Dawkins (2006) has called it, is that groups may develop strategies for punishing free riders, so that they cannot outcompete the altruists. Moreover, it should also be noted that Hamilton’s (1964a, 1964b) theory of inclusive fitness, often invoked in order to explain altruistic behavior in a way that supports the idea that selection cannot operate at the level of groups, actually may be more consistent with the group selection idea. Indeed, Hamilton later gave up on the idea that altruism could only be adaptive when the individual that benefits shares genes that are “identical by descent”. This is how he puts it in his (1975) paper on the *Innate social aptitudes of man*: “Because of the way it was first explained, the approach using inclusive fitness has often been identified with ‘kin selection’ as a way of establishing altruistic social behavior by natural selection […] But the foregoing discussion shows that kinship should be considered just one way of getting positive regression of genotype in the recipient, and that it is this positive regression that is vitally necessary for altruism. Thus the inclusive-fitness concept is more general than ‘kin-selection’.” Furthermore, in some of his less known work, Hamilton (1987/2001) also uses his theory of inclusive fitness to explain phenomena such as groupish nepotism, and he offers some interesting reflections on why he initially included the clause about genes having to be “identical by descent”.

27 For a more detailed description of the patterns of reasoning used by evolutionary psychologists, see Lukaszewski, Lewis et al. (2020).

28 Stewart-Williams (2018, 290–291) has aptly noted that there is a sense in which *any* scientific hypothesis is a just-so-story before it has been tested, but that this sort of objection seldom is levelled at other fields.

29 Compare, e.g., the hypothesis about sex differences in romantic jealousy, and Kanazawa’s hypothesis about cognitive ability, which predicts that it should be correlated with “evolutionarily novel” behaviors in general—both of which are discussed above.
